# Acetylcholine Provocation Testing for Coronary Vasomotor Disorders

**DOI:** 10.31083/RCM52151

**Published:** 2026-09-16

**Authors:** Andrea Caffè, Francesco M. Animati, Rocco A. Montone

**Affiliations:** ^1^Department of Cardiovascular and Pulmonary Sciences, Catholic University of the Sacred Heart, 00168 Rome, Italy; ^2^Department of Cardiovascular Medicine, Fondazione Policlinico Universitario A. Gemelli IRCCS, 00168 Rome, Italy

**Keywords:** acetylcholine provocation testing, coronary vasospasm, ANOCA, MINOCA, angina pectoris, angina pectoris, variant, microvascular angina, coronary microvascular dysfunction, invasive coronary function testing

## Abstract

Vasospastic angina and microvascular angina are increasingly recognized as important causes of myocardial ischemia in patients with angina or myocardial infarction and non-obstructive coronary arteries (ANOCA/MINOCA). Unlike obstructive coronary artery disease, these conditions result from dynamic alterations in epicardial and/or microvascular tone that are not detected by standard coronary angiography. Intracoronary acetylcholine (ACh) provocation testing enables direct assessment of coronary vasomotor function by evaluating both endothelial integrity and smooth muscle reactivity. Moreover, ACh can identify epicardial spasm, microvascular spasm, or impaired endothelium-dependent vasodilation, thereby providing precise characterization of the underlying pathophysiological mechanism. When performed using standardized protocols, ACh testing has shown a high diagnostic yield and a reassuring safety profile. In addition to the associated diagnostic relevance, the ACh response carries prognostic significance, as abnormal findings are associated with a higher risk of recurrent angina and adverse cardiovascular events. Furthermore, randomized data demonstrated that treatment tailored to invasive coronary function testing improves symptoms and quality of life. This review summarizes the pathophysiological background, practical aspects, safety, and clinical value of ACh provocation testing, underscoring its central role in a more personalized approach to patients with ANOCA/MINOCA.

## 1. Introduction

Coronary vasomotor disorders represent a clinically significant and mechanistically distinct group of conditions underlying myocardial ischemia in the absence of obstructive coronary artery disease [1]. These disorders encompass vasospastic angina (VSA) and microvascular angina (MVA) attributable to microvascular spasm, which constitute pathophysiological mechanisms underlying specific clinical phenotypes of angina or myocardial infarction with non-obstructive coronary arteries (ANOCA/MINOCA) [2,3,4]. VSA is characterized by hyperreactivity of the epicardial coronary arteries, resulting in vasoconstriction leading to a transient compromise of coronary blood flow (CBF), whereas MVA reflects dysfunction of the coronary microcirculation, which may manifest as microvascular spasm - a form of coronary vasoconstriction disorder - or as increased microvascular resistance and impaired vasodilatory reserve, mechanisms not primarily related to abnormal vasomotion [1,2].

Unlike obstructive coronary artery disease (CAD), coronary vasomotor disorders are driven by dynamic changes of epicardial and/or microvascular coronary tone [2]. These abnormalities are highly prevalent among patients with ANOCA or MINOCA [5,6,7,8,9], and frequently remain underdiagnosed without dedicated coronary vasomotor function testing (CFT). If left unrecognized, they may lead to persistent anginal symptoms, impaired quality of life (QoL), and adverse clinical outcomes, including malignant arrhythmias, recurrent acute coronary syndromes (ACS), and hospitalizations often leading to repeat coronary angiography [7,10,11,12]. Contemporary guidelines emphasize the importance of comprehensive physiologic assessment - including CFT with intracoronary acetylcholine (ACh) provocation testing - to identify specific vasomotor endotypes and guide mechanism-based therapy, reflecting their prognostic and therapeutic relevance in chronic and acute coronary syndromes [13] (Fig. 1).

**Fig. 1.**
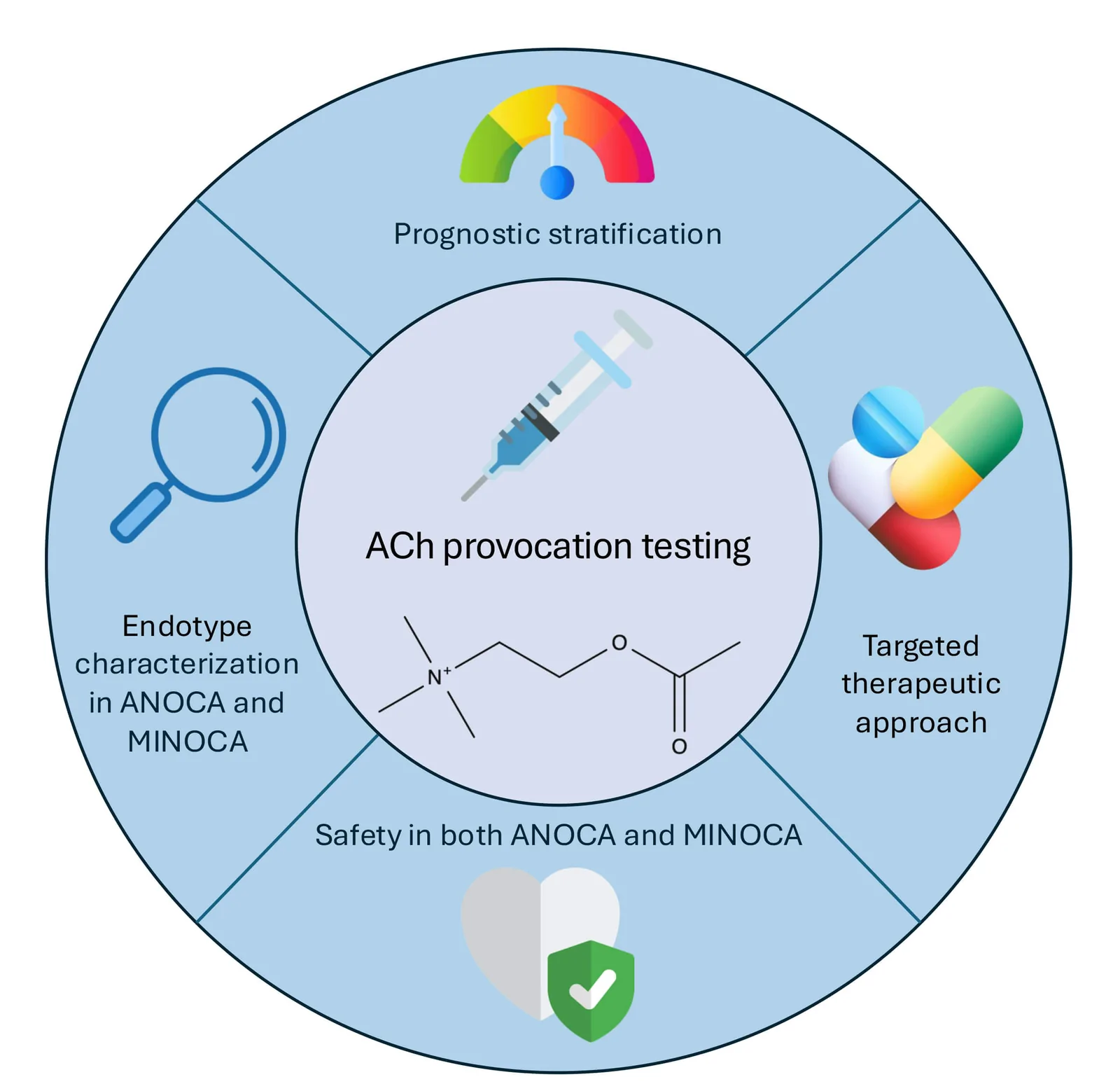
**Clinical applications of acetylcholine provocation testing**. Intracoronary ACh testing allows refined pathophysiological characterization of patients with ANOCA and MINOCA, offers incremental prognostic stratification, and informs mechanism-based therapy, with a reassuring safety profile. ACh, acetylcholine; ANOCA, angina with non-obstructive coronary arteries; MINOCA, myocardial infarction with non-obstructive coronary arteries. Created in Flaticon.

Coronary angiography is limited to the assessment of the epicardial luminogram, not evaluating vasomotor abnormalities and microvascular function, thus leading to underdiagnosis of clinically relevant ischemic mechanisms in patients with non-obstructive coronary arteries, suboptimal management, and inappropriate patient reassurance [14,15,16]. Invasive CFT has emerged as a key approach to overcome these boundaries by enabling direct assessment of coronary vasomotor function [17,18,19]. Specifically, ACh provocation testing offers a high diagnostic yield, guiding individualized pharmacological therapy, improving symptom burden, and providing valuable prognostic information [7,20,21,22,23].

In 1988, Okumura and Yasue [24] demonstrated that intracoronary ACh provocation testing provided high diagnostic accuracy for VSA, reporting elevated sensitivity (90%) and specificity (99%). Since then, ACh testing has been increasingly refined and is currently regarded as a generally safe invasive approach, despite the persistence of substantial heterogeneity in dosing regimens across centres [17,22]. Nevertheless, its clinical adoption remains limited, largely due to persistent concerns regarding procedural safety, which confine its use to specialized centres and a minority of patients with non-obstructive CAD [14].

This review aims to provide a comprehensive overview of the pathophysiological rationale, methodology, and clinical implications of ACh provocation testing, emphasizing its role in pathophysiology-guided, personalized patient management within modern invasive diagnostic strategies for coronary vasomotor disorders.

## 2. Coronary Vasomotor Disorders: Pathophysiological Background

### 2.1 Normal Coronary Vasomotion

Coronary vasomotion is regulated by the endothelium through the balanced release of vasodilator and vasoconstrictor mediators (Fig. 2). Endothelium-dependent vasodilation is mediated by nitric oxide (NO), prostacyclin, and endothelium-derived hyperpolarizing factors (EDHFs), whereas endothelin-1 (ET-1) represents a vasoconstrictor pathway [25]. The contribution of these mediators varies along the coronary circulation: NO predominantly regulates epicardial coronary artery tone, while EDHFs are the main determinants of endothelium-dependent vasodilation at the microvascular level [25,26]. Physiological endothelial stimulation induces vasodilation and increases CBF, whereas endothelial dysfunction is characterized by a reduced vasodilatory response or paradoxical vasoconstriction due to impaired release or bioavailability of endothelial mediators [25]. In addition, abnormal coronary vasomotion may arise from endothelium-independent mechanisms, primarily related to enhanced vascular smooth muscle cell (VSMC) reactivity to constrictor stimuli [27,28].

**Fig. 2.**
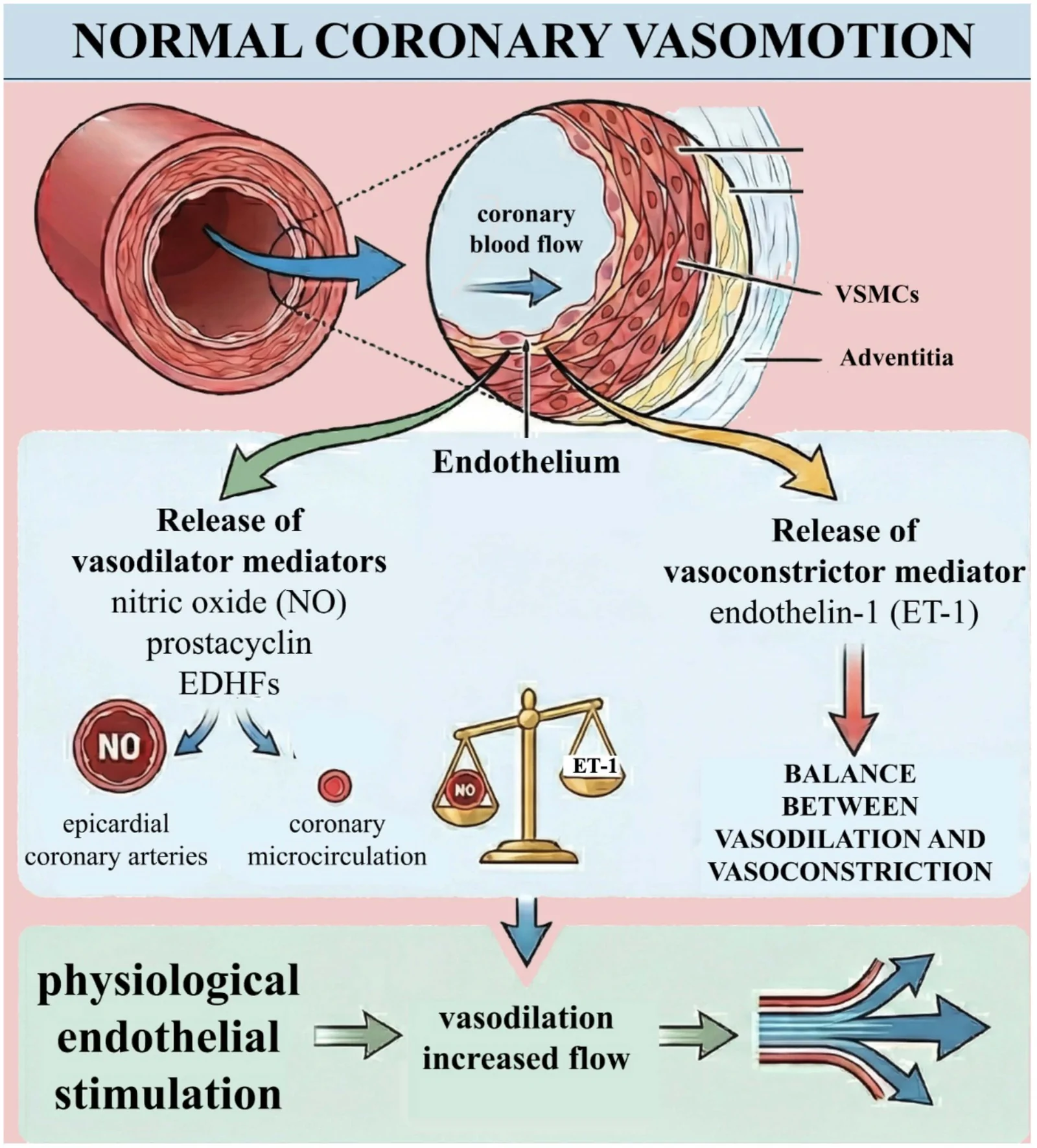
**Physiology of normal coronary vasomotion**. Physiological stimuli (shear stress and metabolic demand) trigger endothelial release of vasodilators (NO, prostacyclin, EDHFs) and vasoconstrictors (e.g., ET-1), whose dynamic balance regulates coronary tone and ensures adequate myocardial perfusion. NO, nitric oxide; EDHFs, endothelium-derived hyperpolarizing factors; VSMC, vascular smooth muscle cell. Created in Flaticon.

### 2.2 Pathophysiology of CAS

A key mechanism underlying coronary artery spasm is VSMC hyperreactivity, characterized by an exaggerated contractile response to vasoconstrictor stimuli such as ACh, serotonin, and catecholamines [27] (Fig. 3). Endothelial dysfunction further modulates this process by reducing NO bioavailability and shifting the vasomotor balance toward vasoconstriction [27]. Enhanced Rho-kinase activity has emerged as a molecular pathway promoting calcium sensitization and sustained vasoconstriction [29]. The autonomic nervous system also plays a relevant modulatory role, with enhanced parasympathetic activity implicated in spasm initiation, particularly at rest and during nocturnal or early morning hours [30]. Furthermore, local inflammatory processes - especially involving perivascular adipose tissue (PVAT) - may contribute to spasm susceptibility by promoting upregulation of Rho-kinase activity and enhancing VSMC hyperreactivity [29,31,32]. Environmental factors (smoking, air pollution exposure), chronic inflammation, oxidative stress, ethnicity (higher CAS prevalence in Japanese cohorts), and genetic polymorphisms also predispose to CAS [33,34,35,36,37,38].

**Fig. 3.**
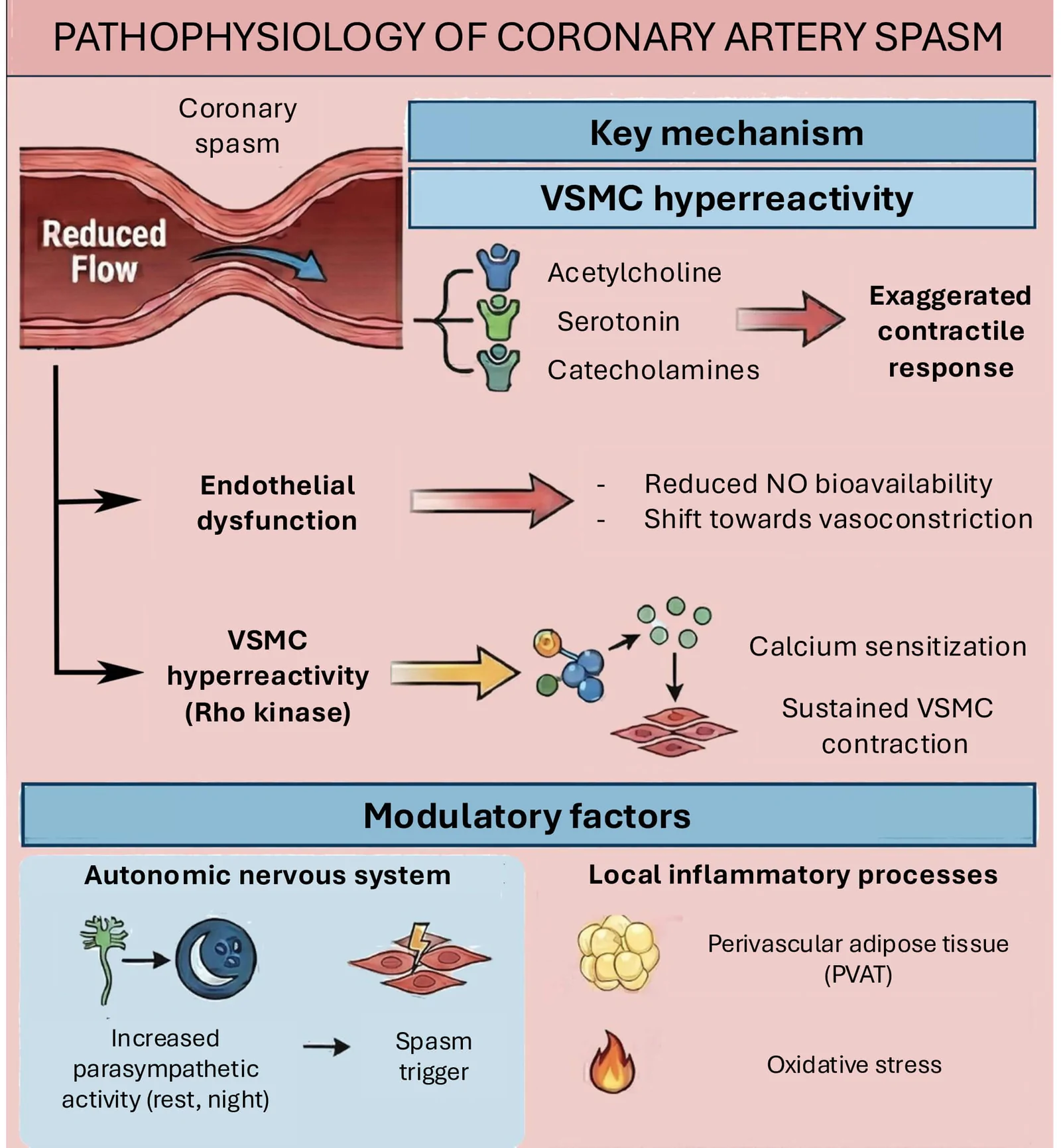
**Pathophysiology of coronary artery spasm**. Enhanced reactivity of VSMCs leads to exaggerated vasoconstriction in response to physiological or minimal stimuli, with a contributory imbalance between vasodilator and vasoconstrictor factors and impaired endothelial modulation. NO, nitric oxide; VSMC, vascular smooth muscle cell. Created in Flaticon.

Myocardial bridging has emerged as a relevant anatomical substrate favoring CAS [39]. The mechanical forces generated by repetitive systolic compression of the bridged segment cause abnormal shear stress patterns that disrupt local endothelial function and lead to heightened vasomotor reactivity [40]. Accumulating clinical evidence demonstrates that patients with myocardial bridging and non-obstructive CAD exhibit a significantly higher susceptibility to ACh-induced epicardial CAS compared to those without a bridge, across both acute and chronic clinical presentations, with total bridged length and percent systolic coronary compression identified as predictors of inducible spasm [41,42,43]. Of interest, endothelial dysfunction may not only co-localize with the bridged epicardial segment, but also extend its pathophysiological impact downstream to the coronary microcirculation [44].

### 2.3 Coronary Microvascular Dysfunction and Pathophysiological Endotypes in ANOCA

Coronary microvascular dysfunction (CMD) encompasses a heterogeneous spectrum of abnormalities affecting the coronary microcirculation, which cannot be assessed by conventional angiography [45]. CMD may be structural, involving capillary rarefaction or microvascular remodeling, or functional, characterized by impaired vasomotor regulation in the absence of fixed vascular changes [45]. From a functional standpoint, two principal endotypes are recognized: impaired microvascular vasodilation and enhanced vasoconstriction (Fig. 4). Impaired vasodilation may result from endothelium-dependent mechanisms, such as reduced NO bioavailability and endothelial dysfunction, or from endothelium-independent abnormalities, reflecting impaired VSMC relaxation and attenuated responses to direct vasodilators such as adenosine [46]. Enhanced vasoconstriction encompasses microvascular spasm as well as a heightened vasoconstrictor milieu, potentially related to increased ET-1 activity, augmented smooth muscle sensitivity to constrictor stimuli, or sympathetic overactivity [46]. In the normal coronary circulation, low-dose ACh induces microvascular vasodilation and an increase in CBF through endothelial NO-mediated mechanisms [47]. In contrast, microvascular spasm reflects an endothelium-independent mechanism predominantly related to hyperreactivity of microvascular smooth muscle cells, resulting in inappropriate constriction of resistance vessels [48].

**Fig. 4.**
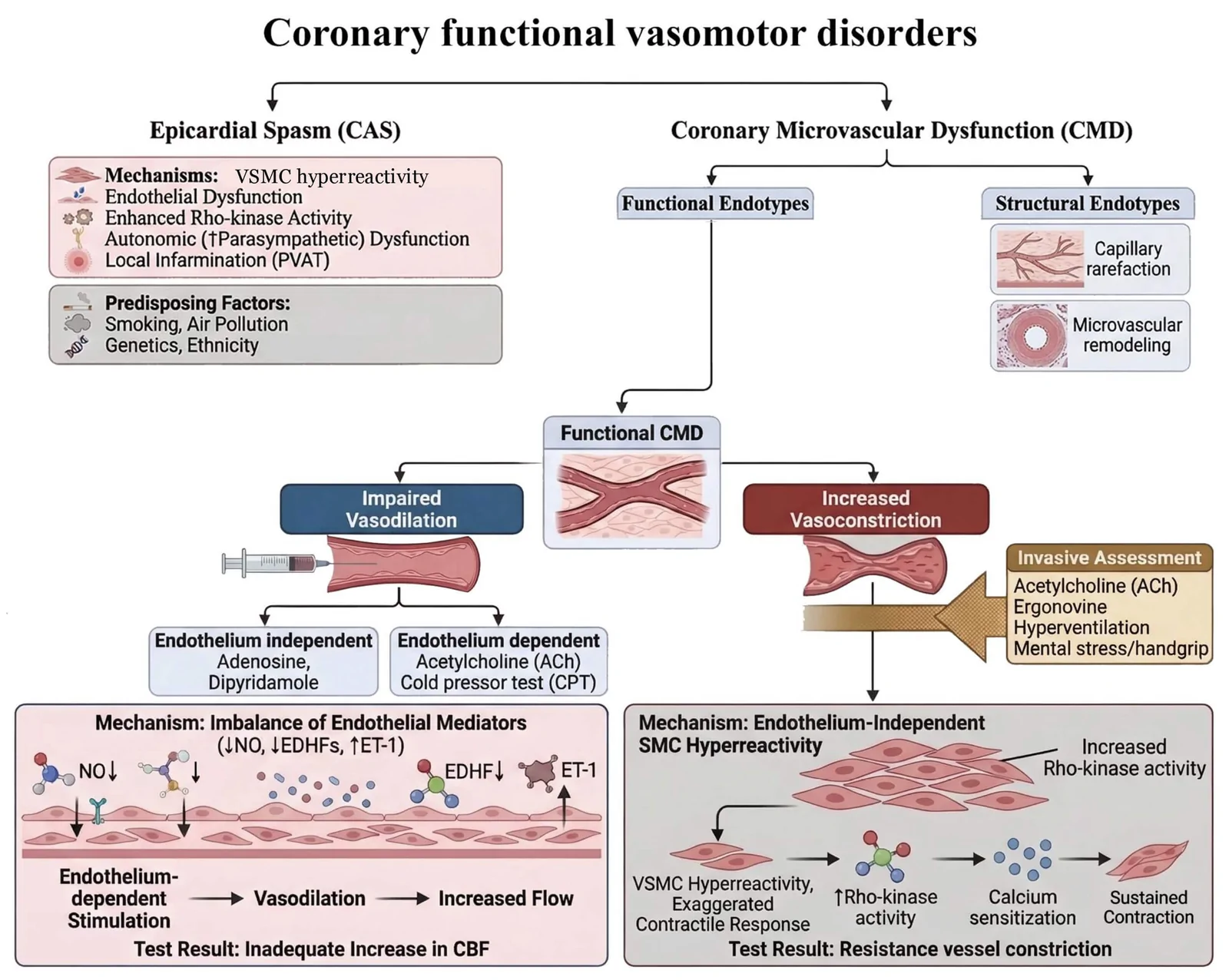
**Hallmarks of coronary functional vasomotor disorders**. ACh provocation testing plays a key role in the assessment of coronary vasomotor disorders, identifying epicardial spasm, functional CMD due to increased vasoconstriction (microvascular spasm), and endothelium-dependent CMD characterized by impaired vasodilation. CBF, coronary blood flow; CMD, coronary microvascular dysfunction; EDHFs, endothelium-derived hyperpolarizing factors; ET-1, endothelin-1; NO, nitric oxide; PVAT, perivascular adipose tissue; SMC, smooth muscle cell; VSMC, vascular smooth muscle cell. Created in Flaticon.

To properly characterize these profiles, contemporary guidelines [13] recommend a comprehensive invasive approach that evaluates both endothelium-dependent vasodilation and vasoreactivity (via ACh testing), and non-endothelium-dependent microvascular function (via coronary flow reserve [CFR] and index of microcirculatory resistance [IMR] measurements, usually via adenosine administration) [49,50]. A recent prospective multicentre study [51] categorized 1001 ANOCA patients into 8 distinct pathophysiological endotypes applying a comprehensive wire-based approach with intracoronary thermodilution, assessing changes in hemodynamic parameters, symptoms, and electrocardiogram (ECG) in response to ACh and adenosine stimulation (Table 1).

**Table 1.** **Endotype classification in ANOCA according to ACh and adenosine testing**.

Endotype definition	Hemodynamic, symptomatic, and ECG characteristics
Epicardial spasm	ACh-Pd/Pa <0.80; >90% epicardial vasoconstriction; angina; ECG modifications
Microvascular spasm	ACh-IMR > rest-IMR; angina; ECG modifications
Endothelial dysfunction	ACh-CFR <1.5; adenosine-CFR >2.5
Ischemia without measurable hemodynamic changes	Angina and ECG modifications during ACh testing without hemodynamic changes
High resting coronary blood flow	Adenosine-CFR <2.5; adenosine-IMR <25
High resistance	Adenosine-CFR <2.5; adenosine-IMR >25
Compensated high resistance	Adenosine-CFR >2.5; adenosine-IMR >25
Enhanced cardiac nociception	Angina without ECG or hemodynamic changes

ACh, acetylcholine; ANOCA, angina with non-obstructive coronary arteries; CFR, coronary flow reserve; ECG, electrocardiogram; IMR, index of microcirculatory resistance.

Importantly, CMD exhibits marked sex-specific differences. Women are disproportionately affected, a phenomenon attributed to hormonal influences, differences in endothelial function, and heightened microvascular sensitivity to vasomotor stimuli [1,52]. Oestrogen deficiency, particularly after menopause, has been associated with reduced NO availability and increased susceptibility to CMD [1,53]. In a large European cohort undergoing ACh provocation testing, female sex was independently associated with both microvascular and epicardial vasospasm, with vasomotor abnormalities occurring at lower ACh doses compared with men [52]. Women often present with coronary vasomotor disorders in the absence of significant atherosclerotic disease and, in many cases, without major conventional risk factors [54], frequently exhibiting diffuse rather than focal epicardial spasm patterns [55]. Data from the Women’s Ischemia Syndrome Evaluation (WISE) study suggest that hormonal status and traditional cardiovascular risk factors account for only a limited proportion of sex-variability, pointing to a potential role of inflammation and autoimmune diseases [56].

## 3. Rationale for ACh Provocation Testing

Under physiological conditions, low-dose ACh induces coronary vasodilation through activation of endothelial muscarinic receptors and subsequent NO release [57,58]. Conversely, in the presence of endothelial dysfunction or enhanced VSMC reactivity, ACh predominantly stimulates muscarinic receptors on VSMCs, resulting in paradoxical vasoconstriction [57,59]. Clinical observations further support this pathophysiological link, as episodes of CAS frequently occur during periods of increased vagal tone, such as at night or early in the morning [57]. By exploiting this differential vascular response, ACh provocation testing enables the functional interrogation of endothelial integrity and coronary hyperreactivity, providing a mechanistic basis for the diagnosis of epicardial and microvascular spasm [59].

In contrast to ACh, ergonovine (ER) primarily induces coronary vasoconstriction through direct activation of serotonergic (5-HT_2_) receptors on VSMCs, with a less prominent dependence on endothelial function [17]. Although ER may stimulate the release of vasodilatory prostanoids from an intact endothelium, this protective mechanism is often attenuated in the presence of endothelial dysfunction, thereby favouring vasospasm in susceptible coronary segments [17]. Clinical studies have reported comparable rates of epicardial CAS induced by ER and ACh [60,61,62]. However, direct comparative investigations have highlighted significant qualitative differences: ER more frequently provokes focal, proximal spasms, whereas ACh tends to elicit diffuse and distal vasoconstriction and, importantly, allows concomitant assessment of microvascular spasm [60]. Furthermore, ACh has shown greater diagnostic sensitivity in specific populations, particularly women [52,63], reinforcing its position as the preferred first-line provocative agent, whereas ergonovine may be reserved for selected cases or employed when ACh testing is negative despite high clinical suspicion [17]. Indeed, a striking sex-specific response has been highlighted, with ACh achieving a diagnostic sensitivity of up to 96.7% in women compared to only 32.8% for ergonovine [63].

## 4. Indications and Patient Selection

### 4.1 Clinical Indications

From a clinical standpoint, invasive CFT with intracoronary ACh provocation testing is indicated in patients with clinical manifestations suggestive of myocardial ischemia in whom obstructive CAD has been ruled out or does not explain the clinical presentation. Candidates belong to two main clinical scenarios: patients with Ischemia with Non-Obstructive Coronary Arteries (INOCA), including those with a high clinical suspicion of VSA [13,64], and those presenting MINOCA and a suspected coronary vasoconstriction disorder, especially in the absence of other ascertained pathophysiological mechanism [65,66].

According to the Coronary Vasomotion Disorders International Study (COVADIS) Group, intracoronary provocative testing is Class I indicated in patients with suspected VSA without documented spontaneous episodes, particularly in the presence of nitrate-responsive rest angina, marked diurnal variation, rest angina without obstructive CAD, ACS presentation without a culprit lesion, unexplained resuscitated cardiac arrest or syncope with chest pain, and recurrent rest angina after successful percutaneous coronary intervention [64]. Furthermore, it is Class IIa recommended in patients with a prior non-invasive diagnosis of coronary spasm who remain symptomatic despite medical therapy [64].

Contemporary international guidelines increasingly acknowledge the clinical utility of invasive CFT - including ACh provocation - for the evaluation of patients with suspected vasomotor disorders, particularly in the context of ANOCA (Table 2). The 2024 ESC Guidelines on Chronic Coronary Syndromes (CCS) now provide a Class I, Level of Evidence (LoE) B recommendation for invasive CFT as part of the diagnostic work-up in patients with persistently symptomatic non-obstructive CAD or uncertain diagnosis after non-invasive evaluation to guide mechanism-based therapy and improve QoL [13]. Importantly, the same document provides a Class I, LoE C recommendation for invasive CFT in patients with suspected vasospastic angina who experience recurrent rest angina associated with transient ST-segment changes that resolve with nitrates and/or calcium channel blockers (CCBs) [13].

**Table 2.** **Indications for invasive coronary provocation testing in current guidelines and consensus documents**.

Class of recommendation			Level of evidence	Recommendation for provocative spasm testing
2015 COVADIS group - vasospastic angina				
	Class I		–	It is recommended to perform provocation testing in patients with a history suspected of VSA without a documented spontaneous episode, especially if they present with: • Nitrate-responsive rest angina, and/or • Marked diurnal variation in symptom onset/exercise tolerance, and/or • Rest angina without obstructive CAD • ACS presentation in the absence of a culprit lesion • Unexplained resuscitated cardiac arrest • Unexplained syncope with antecedent chest pain • Recurrent rest angina following angiographically successful PCI.
	Class IIa		–	It should be considered to perform provocation testing in patients who have been diagnosed with CAS by non-invasive evaluation and are unresponsive to drug therapy.
	Class III		–	It is not recommended to perform provocation testing in the setting of emergent ACS, severe fixed multivessel CAD (including LM artery stenosis), severe myocardial dysfunction (Class IIb if symptoms are suggestive of vasospasm), or in patients without any symptoms suggestive of VSA.
2023 ESC - management of ACS (MINOCA)				
	Class I		C	It is recommended that all patients with an initial working diagnosis of MINOCA undergo a structured diagnostic algorithm, including “functional coronary angiography” with assessment of coronary vasoreactivity to identify underlying mechanisms such as epicardial or microvascular spasm.
2024 ESC - CCS				
	Class I		B	It is recommended to perform invasive CFT as part of the diagnostic work-up in patients with persistently symptomatic non-obstructive CAD or uncertain diagnosis after non-invasive evaluation to guide mechanism-based therapy and improve quality of life.
	Class I		C	It is recommended to perform invasive CFT in patients with suspected VSA who experience recurrent rest angina associated with transient ST-segment changes that resolve with nitrates and/or CCBs.
JCS Guidelines (VSA, MINOCA, and INOCA) (2023 Update)				
	VSA			
		Class I	B	It is recommended to perform pharmacological CAS provocation tests for patients with suspected VSA based on symptoms but not diagnosed with CAS by noninvasive evaluation.
		Class IIa	B	Pharmacological CAS provocation tests should be considered for patients diagnosed with CAS by noninvasive evaluation and for whom the effect of medical therapy is inconclusive or inadequate.
		Class IIb	B	Pharmacological CAS provocation tests may be considered for patients who have been diagnosed with CAS by noninvasive evaluation and in whom medical treatment has been effective.
		Class III (No benefit)	C	It is not recommended to perform pharmacological CAS provocation tests for patients without symptoms suggestive of VSA.
		Class III (Harm)	C	Pharmacological coronary spasm provocation tests should not be performed for patients with ACS for which coronary angiography is performed and in whom serious complications from induced CAS are expected.
	MINOCA			
		Class IIb	C	CAS provocation test may be considered in the acute setting for patients in whom alternative conditions, such as plaque disruption and SCAD, have been definitely ruled out, under conditions in which adequate safety considerations have been taken into account.
		Class III (No benefit)	C	CAS provocation test is not recommended in patients with documented attacks, such as spontaneous or nitroglycerin-induced remission of ST-segment elevation on ECG or occlusive or severely stenotic lesions on coronary angiography.
		Class III (Harm)	C	CAS provocation test should not be performed in the acute phase in patients in whom drug-induced CAS is expected to cause severe complications (e.g., patients with severely depressed cardiac function, congestive heart failure, etc.).
	INOCA			
		Class IIa	C	In patients with angina pectoris and demonstrated myocardial ischemia but no significant stenosis of the epicardial coronary arteries, a pharmacological CAS provocation test should be considered to confirm the presence of coronary microvascular spasm during coronary angiography.

ACS, acute coronary syndrome; CAD, coronary artery disease; CAS, coronary artery spasm; CCBs, calcium channel blockers; CCS, chronic coronary syndrome; CFT, coronary function testing; COVADIS, Coronary Vasomotor Disorders International Study group; ECG, electrocardiogram; ESC, European Society of Cardiology; INOCA, ischemia with non-obstructive coronary arteries; JCS, Japanese Circulation Society; LM, left main; MINOCA, myocardial infarction with non-obstructive coronary arteries; PCI, percutaneous coronary intervention; SCAD, spontaneous coronary artery dissection; VSA, vasospastic angina.

Moreover, the 2023 ESC Guidelines for the management of ACS recommend that all patients with an initial working diagnosis of MINOCA undergo a structured diagnostic algorithm (Class I, LoE C), including “functional coronary angiography” with assessment of coronary vasoreactivity to identify underlying mechanisms such as epicardial or microvascular spasm [65].

Recently, acute presentation with MINOCA, myocardial bridging, elevated C-reactive protein, and dyslipidaemia have been identified as independent predictors of a positive ACh response, forming a simple four-component ABCD score that may assist in identifying patients with a higher pre-test probability of vasomotor dysfunction [67]. Such an approach may facilitate clinical decision-making and support more appropriate management strategies.

### 4.2 Contraindications and Precautions

According to the COVADIS group [64], intracoronary provocative testing is not recommended (Class III) in patients with emergent ACS, severe fixed multivessel or left main CAD, severe myocardial dysfunction, and in patients without clinical features suggestive of VSA. However, invasive coronary provocative testing has been shown to be feasible and safe in patients presenting with ACS and suspected vasomotor abnormalities, without a significantly higher complication rate compared with elective testing [7,68,69]. The 2023 JCS focused update on Diagnosis and Treatment of VSA and CMD recommends performing invasive coronary provocative testing in an acute setting only after exclusion of alternative causes of ACS (e.g., plaque instability or spontaneous coronary artery dissection) and advises against its use in patients presenting with ACS and concomitant heart failure [66].

## 5. Methodology of ACh Provocation Testing

Intracoronary ACh provocation testing should be performed in a fully equipped catheterization laboratory with immediate availability of intracoronary nitroglycerine (NTG), resuscitation equipment, and defibrillator [70]. Continuous 12-lead ECG and invasive haemodynamic monitoring are mandatory throughout the procedure [71].

ACh testing may be significantly influenced by the concomitant or recent use of vasoactive medications. Accordingly, most protocols recommend discontinuation of cardiovascular drugs with vasomotor effects 24 to 72 hours prior to CFT to avoid attenuation of vasospastic responses [71]. Some centres routinely administer intra-arterial radial “cocktails” containing CCBs with or without nitrates, which may blunt coronary vasospasm during ACh testing [2]. Regardless of the chosen access site, a 6 Fr system is recommended to ensure procedural safety and standardized coronary infusions [2].

All ACh testing protocols consistently include the administration of 200–300 µg of intracoronary NTG after the final ACh dose, or after provocation of significant coronary spasm, to promptly reverse vasoconstriction and restore baseline coronary tone [71].

According to the original method proposed by Okumura and Yasue [24], ACh is first administered into the left coronary artery (LCA) in incremental doses (20, 50, and 100 μg) and subsequently into the right coronary artery (RCA) (20 and 50 μg) over 20 seconds, with a 5-minute interval between injections (Fig. 5). Sueda et al. [72] further explored higher maximal doses of 200 μg for the LCA and 80 μg for the RCA. Coronary angiography is performed either upon the occurrence of chest pain, ST-segment changes, or 1–2 minutes after each injection, and finally following intracoronary nitrate administration. This protocol - except for the maximum dosages - has been incorporated into the most recent JCS Guidelines on the Diagnosis and Treatment of VSA and CMD [66].

**Fig. 5.**
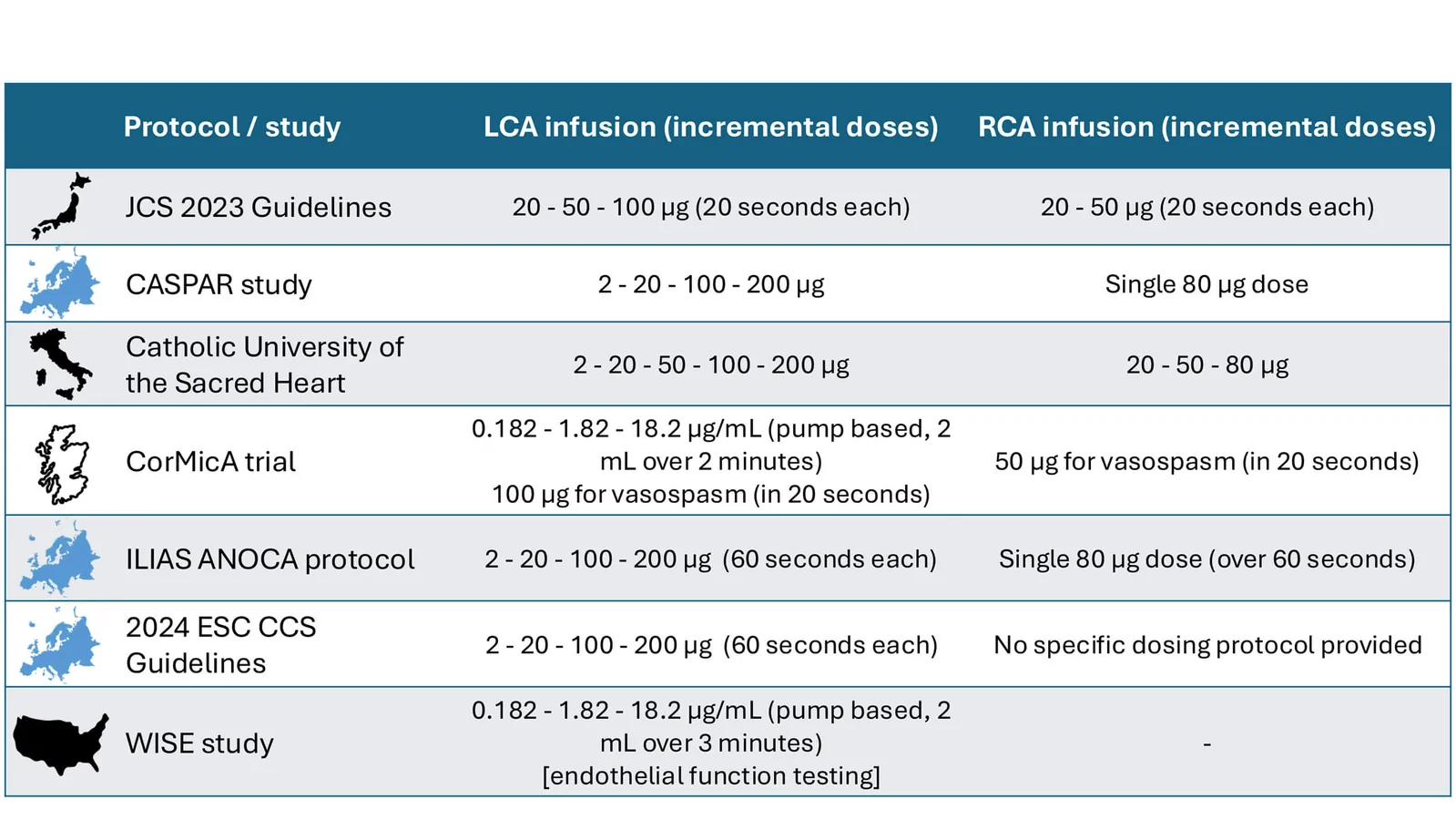
**Overview of ACh provocation testing protocols reported in the literature**. The infusion time for each ACh dose is 2–3 minutes, unless otherwise specified. ACh, acetylcholine; CASPAR, Coronary Artery Spasm as a Frequent Cause for Acute Coronary Syndrome; CorMicA, Coronary Microvascular Angina; ILIAS ANOCA, Inclusive Invasive Physiological Assessment in Angina Syndromes - Angina with Non-Obstructive Coronary Artery Disease; JCS, Japanese Circulation Society; LCA, left coronary artery; RCA, right coronary artery; WISE, Women’s Ischemia Syndrome Evaluation; CCS, Chronic Coronary Syndromes; ESC, European Society of Cardiology. Created in Flaticon.

In Europe, the protocol described by Ong et al. in the CASPAR study [6] is commonly used, involving incremental doses of 2, 20, 100, and 200 μg of ACh infused manually into the LCA over 3 minutes, and a single dose of 80 μg into the RCA. At the Catholic University of the Sacred Heart, we adopted a similar approach, administering ACh in incremental doses of 2, 20, 50, 100, and 200 μg gradually infused into the LCA, and 20, 50, and 80 μg into the RCA [7].

Intracoronary ACh may be administered using a mechanical infusion pump (1 mL/min over 2 minutes), enhancing procedural standardization, although manual injection remains straightforward and widely practiced, with a comparable diagnostic yield [20,73]. Notably, the CorMicA trial adopted a pump-based protocol, assessing endothelial function through sequential 2-minute infusions of ACh at increasing concentrations (10^–6^, 10^–5^, and 10^–4^ mol/L), followed by a bolus injection (100 μg in the LCA or 50 μg in the RCA) to evaluate inducible vasospasm [8,20].

Recently, in the ILIAS ANOCA protocol [21], ACh provocation was performed by manual intracoronary administration of incremental doses (2, 20, 100, and 200 μg) infused over approximately 60 seconds into the LCA. If the test was negative, the RCA was subsequently assessed with a single 80 μg dose administered over 60 seconds [21].

In most protocols, the RCA is not routinely assessed, or testing is discontinued if spasm is already induced in the LCA, mainly because ACh administration in the RCA may provoke transient atrioventricular block [71]. Routine RCA testing is specifically recommended by the JCS guidelines [74], while other protocols reserve RCA evaluation for selected cases, such as when the clinical presentation raises suspicion of right coronary involvement [71].

## 6. Diagnostic Criteria and Interpretation

### 6.1 Standardized Diagnostic Frameworks for Epicardial and Microvascular Coronary Spasm

Diagnostic interpretation of ACh provocation testing is based on an integrated evaluation of symptoms, electrocardiographic changes, angiographic findings, and, when adopted, invasive coronary physiological measurements [1]. According to the COVADIS criteria [64] an invasive provocative test is considered diagnostic for epicardial coronary artery spasm when all of the following are documented in response to the pharmacological stimulus: reproduction of the patient’s typical chest pain, ischemic ECG changes, and angiographic evidence of ≥90% coronary vasoconstriction compared with the reference state after intracoronary NTG administration (Table 3). Within the same diagnostic framework, coronary microvascular spasm is defined by the reproduction of typical symptoms accompanied by ischemic ECG changes in the absence of ≥90% epicardial vasoconstriction [75] (Table 4).

**Table 3.** **COVADIS diagnostic criteria for VSA**.

**Nitrate-responsive angina (≥1 of the following):**	
Rest angina (especially in the night and early morning)	
Marked diurnal variation in exercise tolerance (reduced in morning)	
Episodes precipitated by hyperventilation	
CCBs (but not beta-blockers) suppress episodes	
**Transient ischemic ECG changes during spontaneous episode (any of the following in ≥2 contiguous leads):**	
ST elevation ≥0.1 mV	
ST depression ≥0.1 mV	
New negative U waves	
**CAS (defined as ≥90% vasoconstriction) with angina and ischemic ECG changes:**	
spontaneously	
in response to a provocative stimulus (typically ACh, ergot, or hyperventilation)	

Definitive VSA: nitrate-responsive angina during spontaneous episodes accompanied by either transient ischemic ECG changes or angiographic evidence fulfilling CAS criteria. Suspected VSA: nitrate-responsive angina during spontaneous episodes with equivocal or unavailable transient ischemic ECG changes and equivocal CAS criteria. ACh, acetylcholine; CAS, coronary artery spasm; CCBs, calcium channel blockers; COVADIS, Coronary Vasomotor Disorders International Study group; ECG, electrocardiogram; VSA, vasospastic angina.

**Table 4.** **COVADIS diagnostic criteria for MVA**.

**Symptoms of myocardial ischemia**	
Effort and/or rest angina	
Angina equivalents	
**Absence of obstructive CAD (<50% diameter reduction or FFR >0.80) by**	
CCTA	
ICA	
**Objective evidence of myocardial ischemia**	
Ischemic ECG changes during an episode of chest pain	
Stress-induced chest pain and/or ischemic ECG changes in the presence or absence of transient/reversible abnormal myocardial perfusion and/or wall motion abnormality	
**Evidence of impaired coronary microvascular function**	
Impaired CFR (≤2.0 or ≤2.5)	
Coronary microvascular spasm, defined as reproduction of symptoms, ischemic ECG shifts but no epicardial spasm during ACh testing	
Abnormal coronary microvascular resistance indices (e.g., IMR >25)	
Coronary slow flow (TIMI frame count >25)	

Definitive MVA: all four diagnostic criteria for MVA met. Suspected MVA: symptoms suggestive of myocardial ischemia (criterion 1) and non-obstructive coronary artery disease (criterion 2), together with either: - objective evidence of myocardial ischemia (criterion 3) - evidence of impaired coronary microvascular function (criterion 4). ACh, acetylcholine; CCTA, coronary computed tomography angiography; ECG, electrocardiogram; CFR, coronary flow reserve; COVADIS, Coronary Vasomotor Disorders International Study group; FFR, fractional flow reserve; ICA, invasive coronary angiography; IMR, index of microcirculatory resistance; TIMI, Thrombolysis in Myocardial Infarction.

It should be acknowledged that several earlier studies adopted less stringent criteria for a positive provocative test [72]. Some investigators classified as diagnostic any transient luminal narrowing >90% associated with either ischemic ECG changes or typical chest pain [76], while others applied lower angiographic cut-offs for epicardial spasm (≥75%), as in the ACOVA and CASPAR study [5,6,61]. Consequently, a proportion of patients previously labelled as having inducible epicardial spasm under these alternative definitions would now be categorized as equivocal according to COVADIS [17]. Furthermore, in the 2023 JCS guidelines update, the diagnostic criteria for coronary spasm were revised and defined as transient total or subtotal focal occlusion (≥90% diameter reduction) of a coronary artery, or ≥90% diffuse vasoconstriction involving at least two contiguous segments, occurring in association with signs or symptoms of myocardial ischemia [66].

A distinction should be made between vasoconstriction and vasospasm. Vasoconstriction refers to the dynamic reduction in vessel calibre compared with the maximal baseline reference state after intracoronary nitrate administration. In ACh testing, a non-diagnostic epicardial coronary diameter reduction (<90%) occurring in the absence of ischemic ECG alterations or typical symptoms represents a continuous pathological response to an otherwise physiological mediator, serving as a marker of endothelial dysfunction [77]. Conversely, epicardial CAS is defined as an exaggerated vasoconstrictor response. It is ascertained only when the vasoconstriction exceeds the angiographic threshold of ≥90% diameter reduction, as established on the basis of haemodynamic evidence indicating that ≥90% coronary obstruction is consistently associated with impairment of resting CBF [74].

### 6.2 Microvascular Spasm: Integration With Pressure/Flow Wire Measurements and the Role of ACh Rechallenge

At present, microvascular spasm is frequently regarded as a tentative diagnosis, established when ACh administration induces angina and ischemic ECG changes in the absence of angiographic evidence of epicardial spasm. Earlier investigations reported indirect markers of myocardial ischemia, such as coronary sinus lactate production and the occurrence of angiographic slow flow during ACh infusion [78,79], without direct assessments of coronary microvascular flow and resistance.

To overcome the inherent limitations of symptom- and ECG-based criteria and to further refine the mechanistic characterization of ACh-induced ischemia, some investigators have proposed integrating pressure- and flow-wire measurements during provocation testing. Wire-based assessment enables simultaneous evaluation of distal coronary pressure (Pd/Pa) [80] and microvascular haemodynamics using thermodilution-derived indices, such as ACh-IMR [81]. In the epicardial compartment, a reduction in Pd/Pa during ACh infusion may provide supportive physiological evidence of functionally relevant vasoconstriction [80]. Conversely, in the absence of epicardial spasm, an increase in IMR during ACh may suggest significant microvascular vasoconstriction, potentially identifying a microvascular spasm phenotype even when conventional ischemic criteria are inconclusive [81].

Furthermore, considering that microvascular spasm may coexist with epicardial spasm, the ACh rechallenge protocol has been proposed to enable an isolated functional assessment of the microcirculation - without the need for wire-based measurements [81,82]. In this approach, if epicardial spasm is induced, intracoronary NTG is administered to resolve epicardial vasoconstriction, and ACh is then re-administered at 100–200 μg [82,83]. The recurrence of angina and/or ischemic ECG changes in the absence of renewed epicardial constriction suggests concomitant microvascular spasm [82,83]. The pathophysiological basis of the ACh rechallenge is represented by the limited responsiveness of the microcirculation to NTG, likely due to reduced biotransformation of the drug in vessels <100 μm in diameter [71]. This pragmatic strategy has also been adopted in the recent ILIAS ANOCA trial [21].

### 6.3 Endothelial Dysfunction Assessment

Graded intracoronary infusion of ACh at lower concentrations (0.182 μg/mL [10^–6^ mol/L] to 18.2 μg/mL [10^–4^ mol/L], typically administered at 1 mL/min) is recommended for the assessment of endothelium-dependent coronary function [1] and has been incorporated into endothelial function testing protocols used in clinical studies [84,85], such as in the Women’s Ischemia Syndrome Evaluation (WISE) study. An abnormal response is defined by either a blunted endothelium-dependent microvascular vasodilatory reaction, conventionally characterized as a <50% increase in resting CBF, or by paradoxical microvascular vasoconstriction with a reduction in CBF [86]. A reduction in flow relative to baseline allows a more sensitive and earlier identification of microvascular spasm, recognizing that flow abnormalities precede ECG changes in the ischemic cascade [70]. Notably, Doppler flow-wire assessment has demonstrated that abnormal ACh-induced CBF responses may be present in approximately one-third of patients with ANOCA despite a preserved adenosine-mediated response [87].

When combined with the wire-based metrics described above [51,88], intracoronary ACh allows a highly refined characterization of vasomotor endotypes. For instance, impaired endothelium-dependent vasodilation can be diagnosed in the presence of an ACh-CFR <1.5 with a preserved adenosine-derived CFR >2.5, thereby excluding a non–endothelium-dependent component of dysfunction [51].

Of interest, it has been observed that ACh can simultaneously induce epicardial vasospasm and microvascular vasodilation in patients with VSA, indicating that epicardial VSMC hyperreactivity and endothelium-dependent microvascular dysfunction may not necessarily coexist [89,90].

## 7. Safety and Complications

Despite its high diagnostic yield, prognostic value, and therapeutic implications, ACh provocation testing remains underutilized in routine clinical practice. This reluctance is largely driven by persistent concerns regarding procedural safety, limited familiarity with standardized protocols, and the perception that the test should be confined to highly specialized centres [14].

We demonstrated the safety and the prognostic relevance of ACh provocation testing in a large cohort study involving both MINOCA and ANOCA patients [22]. Transient bradyarrhythmias and hypotension represent the most frequently observed adverse events during ACh provocation testing [91]. However, bradycardia is often underreported in the literature, as it largely reflects the expected physiological effect of ACh on cardiac conduction system and may not be apparent in patients undergoing upfront backup pacing or those with a baseline paced rhythm [22,91]. Importantly, clinically relevant bradyarrhythmias - defined as symptomatic episodes or events requiring pharmacological or pacing intervention - are uncommon, with an incidence reported below 1% in contemporary studies [61].

Major complications associated with ACh testing are rare but include ventricular tachycardia and ventricular fibrillation [22,61,76,91], while minor complications include the occurrence of atrial fibrillation and supraventricular tachycardia [22]. Notably, the incidence of arrhythmic events during ACh provocation testing is comparable to that observed during spontaneous VSA episodes (approximately 7%) [76]. Severe procedure-related complications such as myocardial infarction and cardiogenic shock due to severe vasospasm or left main coronary artery spasm are extremely rare and have been described in isolated reports [68,84]. A meta-analysis including more than 12,000 patients undergoing ACh provocation testing for both ANOCA and MINOCA reported no procedure-related deaths and a major complication rate of approximately 0.5% [91]. Furthermore, ACh dose escalation or the administration of high-dose ACh have not been associated with significantly higher complication rates [22,91,92].

We also demonstrated that complications during ACh testing are more frequently observed in patients with a history of paroxysmal atrial fibrillation and significant left ventricular diastolic dysfunction [22]. Crucially, complication rates have not been shown to differ between patients with positive versus negative ACh provocation test results [22], nor according to clinical presentation (MINOCA vs. ANOCA) or the setting of coronary angiography (emergency vs. non-emergency) [22,68].

Ventricular arrhythmic events may be more closely related to a direct arrhythmogenic effect of ACh rather than being solely a consequence of ACh-induced myocardial ischemia [22]. Supporting this hypothesis, increased baseline QT dispersion has been shown to predispose patients to arrhythmic complications during provocation testing, and ACh-induced transient exacerbation of QT dispersion may further amplify this vulnerability, regardless of overall test positivity [22,93].

### Risk Mitigation Strategies

Careful patient selection, continuous monitoring, and predefined safety measures are essential components of ACh provocation testing. Transient atrioventricular block typically resolves within seconds after slowing the infusion rate or discontinuing ACh administration [71]. Given the risk of bradyarrhythmias, particularly during intracoronary administration of ACh in the RCA, the JCS guidelines recommend the placement of a backup temporary pacing electrode in the right ventricle at the start of the procedure [74].

Of note, ACh must be administered intracoronary due to its short half-life, which allows rapid and controlled reversal of induced spasm with intracoronary NTG, enhancing procedural safety [71]. In contrast, ER can be administered intravenously, but systemic administration may provoke prolonged or simultaneous spasm of both the right and left coronary arteries, which can be more difficult to manage and reverse [71].

## 8. Clinical Impact and Prognostic Implications

ACh provocation testing provides relevant prognostic information. Available evidence indicates that patients with a positive ACh test experience a less favourable clinical course compared with those with a negative response [22,23]. Specifically, ACh testing positivity portends a significantly higher burden of recurrent angina, poorer angina-related QoL, and a higher risk of adverse cardiovascular events during follow-up, driven by hospitalizations for unstable angina [11,22]. Recent evidence suggests that a positive test response at lower ACh doses may be associated with a significantly higher risk of long-term major adverse cardiovascular events [94]. Furthermore, in patients with ANOCA, impaired ACh-CBF response has been associated with a higher incidence of adverse cardiovascular outcomes, suggesting the prognostic significance of endothelium-dependent microvascular function evaluation beyond adenosine-derived CFR assessment [77]. Of relevance, current evidence suggests that complications occurring during intracoronary ACh provocation testing do not appear to be associated with an unfavourable medium- to long-term prognosis [22].

Of importance, we demonstrated for the first time that the prognostic implications of ACh testing appear to be particularly pronounced in the setting of MINOCA, a cohort in which a positive provocation test is a significant predictor of cardiovascular and all-cause mortality [7].

Beyond a dichotomous interpretation of test results, we demonstrated that ACh provocation testing may offer incremental prognostic stratification when individual COVADIS diagnostic criteria are considered [95]. In a cohort including over 500 INOCA and MINOCA patients, we showed that the incidence of major adverse cardiovascular and cerebrovascular events increased progressively according to the number of positive COVADIS criteria components documented during testing [23]. Notably, even when a test is classified as negative or inconclusive due to the absence of symptoms during or ischemic ECG changes during the procedure, the documentation of a significant epicardial coronary artery spasm (≥90% diameter reduction) represents an independent predictor of adverse clinical outcomes [23]. This underscores that vasomotor hyperreactivity and subclinical myocardial ischemia may be associated with substantial risk regardless of symptom reproduction.

Furthermore, in the context of myocardial bridging, an ACh provocative test demonstrating inducible CAS at the site of the bridge predicts a worse angina symptom status and recurrence at follow-up in patients with stable ANOCA [42], whereas it is significantly associated with a higher risk of major adverse cardiovascular events in those presenting with MINOCA [43].

## 9. Therapeutic Implications

The implementation of diagnostic strategies incorporating ACh provocation testing has been shown, in randomized clinical trials, to improve patient-reported QoL at follow-up in both ANOCA [20,21] and MINOCA populations [96]. A structured diagnostic–therapeutic strategy based on invasive CFT has been evaluated in the CorMicA [20] and ILIAS ANOCA [21] trials, enrolling patients with ANOCA. Across these trials, disclosure of invasive CFT results and subsequent mechanism-guided therapy were consistently associated with meaningful improvements in angina-related symptoms and health-related QoL compared with usual care [20,21].

In the PROMISE trial [97], a comprehensive invasive diagnostic strategy incorporating ACh provocation testing was used to identify underlying coronary vasoconstriction abnormalities in patients presenting with MINOCA, and medical therapy was subsequently tailored according to the documented pathophysiological substrate. This personalized strategy was associated with a better angina-related QoL during follow-up [96]. Notably, adverse outcomes in MINOCA patients with a positive ACh test have been linked to suboptimal long-term management, including dose reduction or discontinuation of CCBs, underscoring the clinical relevance of ACh testing not only for risk stratification but also for guiding and reinforcing disease-specific treatment strategies [7,98].

Importantly, identifying CAS at the site of myocardial bridging is clinically relevant to avoid inappropriate medical treatment [43,99]. While beta-blockers represent the first-line therapy to reduce systolic compression in myocardial bridging, they may be detrimental in the presence of vasospasm, as they may exacerbate vasoconstriction through unopposed α-adrenergic stimulation [39]. Consequently, CCBs should be preferred as the primary strategy when a vasomotor disorder is documented [39,43].

## 10. Contemporary Consensus Documents, Current Guidelines and Recommendations

Contemporary guidelines advocate for an integrated invasive assessment in ANOCA, combining anatomical imaging with CFT to evaluate both epicardial and microvascular vascular beds through intracoronary ACh administration [1,2,70].

Lower ACh doses (i.e., 10^–6^, 10^–5^, and 10^–4^ mol/L, equivalent to 0.182 μg/mL, 1.82 μg/mL, and 18.2 μg/mL administered at 1 mL/min) are recommended by the EAPCI Expert Consensus Document on Ischaemia with Non-Obstructive Coronary Arteries [1], whereas the 2024 ESC Guidelines for CCS indicate 60-second injections of low-dose ACh (2–20 µg) to assess endothelium-dependent vasodilation and higher-dose ACh (100–200 µg) to provoke vasospasm [13].

The optimal sequence of endothelium-dependent (ACh) and endothelium-independent (adenosine) testing during CFT remains subject to local expertise [2]. A central issue in determining the sequence is the timing of NTG administration, which is routinely given at the end of ACh testing to reverse vasospasm and to confirm endothelium-independent epicardial vasodilation [2]. Conversely, when adenosine-based assessment is performed first, prophylactic NTG - traditionally administered before pressure or flow wire placement to prevent wire-induced spasm and ensure maximal hyperaemia - should be withheld to avoid masking a subsequent vasospastic response to ACh [2].

Both the 2024 ESC Guidelines for CCS [13] and the 2023 JCS guidelines [66] favour an ACh-first strategy, followed by NTG administration and subsequent endothelium-independent microvascular function assessment using intravenous adenosine. This approach streamlines the protocol by integrating NTG use into the ACh phase and avoiding potential interference with vasospasm provocation testing. However, some protocols employ wire-based adenosine testing first (without prophylactic NTG), followed by ACh and final NTG administration, arguing that ACh-induced spasm could theoretically alter baseline microvascular resistance and affect the accuracy of subsequent adenosine-derived indices [20,51,100]. This strategy aligns with the recommendations of the EAPCI Expert Consensus Document on Ischaemia with Non-Obstructive Coronary Arteries [1].

Overall, current consensus emphasizes that meticulous execution of CFT may be more relevant than the procedural order, provided that NTG is not administered immediately before ACh testing [2].

## 11. Limitations and Controversies

Despite its high diagnostic yield and prognostic and therapeutic value, certain unresolved controversies and practical limitations of ACh testing must be acknowledged to ensure accurate interpretation and to inform efforts toward protocol standardization.

A methodological debate centers on dose escalation and protocol heterogeneity, which significantly hamper inter-study comparability and clinical decision-making [71]. Although some provocation protocols employ ACh doses up to 200 μg in the LCA, whether such dose escalation meaningfully enhances the diagnostic performance of ACh testing remains uncertain. Individual studies have suggested that increasing the maximal ACh dose from 100 to 200 μg results in a higher rate of positive tests and more frequent detection of multivessel coronary spasm [101]. This regimen has also been associated with significantly greater sensitivity, with only a nonsignificant reduction in specificity [102], although data may not yet be sufficient to rule out non-specific epicardial vasoconstriction [66].

Comparisons based solely on the peak ACh dose per injection may be misleading, given its short half-life. Thus, the duration of administration should be taken into account when interpreting test results and comparing different protocols [71]. Contemporary JCS guidelines endorse a rapid ACh administration over 20 seconds [66], generating a transient peak concentration that has been shown to induce more pronounced vasoconstriction, accompanied by a higher frequency of ischemic ECG changes and symptom reproduction, compared with prolonged infusion protocols [103].

At the other end of the spectrum of ACh administration protocols, landmark Western studies have favored more prolonged ACh infusions. For instance, the WISE study [84] employed sequential infusions of low-dose ACh predominantly designed to evaluate endothelial function, a methodology that was subsequently incorporated into the EAPCI Expert Consensus Document [1]. Conversely, the CorMicA trial [20] incorporated this gradual low-dose infusion protocol for endothelial assessment while integrating higher-dose rapid boluses – specifically 20-second administration of 100 μg in the LCA and 50 μg in the RCA – for vasospasm provocation. The 2024 ESC Guidelines for CCS [13], and the ILIAS ANOCA and AID-ANGIO protocols [19,21] also emphasize the role of differential assessment of endothelium-dependent vasodilation and inducible vasospasm, and recommend a 60-second administration duration, positioning this protocol midway between rapid boluses and more prolonged infusion strategies.

Until a broadly accepted benchmark is established, such variations in dosing, administration patterns, and diagnostic criteria directly impact how test results are compared across literature.

From a pragmatic perspective, protocol heterogeneity also involves the potential for tailored ACh dosing sequence to optimize procedure times and contrast volume. Evidence from studies adopting the JCS protocol suggests that avoiding the intermediate 50 μg dose in the LCA is safe and feasible when initial vasoconstriction is minimal (<25%) by 20 μg [104]. Similarly, patients with a negative LCA provocative test exhibit a negligible incidence of RCA spasm (<1%) at the initial 20 μg dose [105]. Therefore, it has been suggested that omitting the initial 20 μg dose in the contralateral artery may streamline the protocol without compromising safety [105].

Although testing of the RCA involves increased procedural time and the risk of transient bradyarrhythmias – avoiding its assessment can compromise overall diagnostic yield. Indeed, in a cohort of patients with a negative ACh test in the LCA, subsequent RCA testing was positive in 8.4% of cases [106]. Provided there is low clinical suspicion of RCA involvement, a strategy restricted to the LCA testing may be reasonable when LCA vasoconstriction remains minimal (<25%) even at high doses (100 μg) [106]. Conversely, RCA evaluation may hold particular relevance in patients with high clinical suspicion based on ischemic ECG modifications, or in those exhibiting intermediate, non-diagnostic LCA vasoconstriction (25–90%) [106]. Therefore, a tailored approach to RCA testing based on both clinical presentation and initial LCA reactivity may optimize the balance between procedural safety and diagnostic performance. Omitting RCA testing, however, risks neglecting multivessel spasm, a recognized marker of worse prognosis [10].

Beyond standardization issues, ACh testing assessment is hindered by real-world considerations. The interpretation of provocative testing based solely on symptoms and ECG changes may be limited in certain clinical scenarios, such as minimal or absent surface ECG abnormalities, ST-segment cancellation due to simultaneous ischemia in different coronary territories, or baseline ECG abnormalities [71]. In this context, the integration of invasive pressure- and flow-wire–based measurements during ACh testing may provide incremental diagnostic information [51]. Furthermore, potential inter-observer variability in angiographic assessment should be considered, particularly when evaluating diffuse, borderline epicardial spasm.

In contemporary practice, invasive CFT remains largely restricted to specialized tertiary centers. Besides persistent concerns regarding potential procedural complications, the progressive implementation of these procedures into routine clinical workflows is limited by heterogeneity in available testing protocols, which often discourages clinicians due to a lack of familiarity. Finally, while the CorMicA and ILIAS ANOCA trials have proven that mechanism-guided therapy through CFT significantly improves health-related QoL and angina status in ANOCA [20,21], its impact on hard clinical outcomes remains to be definitively established in large-scale prospective studies [107].

## 12. Future Perspectives

Future research should aim to standardize ACh provocation protocols across centres, harmonizing dosing regimens, infusion times, and diagnostic criteria, in order to enhance inter-study comparability. The integration of comprehensive wire-based physiological assessment - including ACh-derived Pd/Pa, IMR, and CFR - has been recently adopted to allow a more precise endotype-based classification of vasomotor disorders [51], refining mechanistic understanding with potential clinical implications. In this regard, separating the dual purposes of the test by utilizing prolonged low-dose infusions for endothelial function assessment and rapid injections for vasospasm provocation could represent a pragmatic pathway to bridge long-standing geographical discrepancies in ACh testing protocol designs [108]. While such a dual-intent framework could reduce procedural and geographical heterogeneity if widely adopted, the complementary implementation of wire-based physiological assessment - which is already required for an accurate evaluation of the endothelial response to ACh - could emerge as a key strategy to provide objective metrics and improve inter-center diagnostic consistency for vasospasm provocation testing execution and interpretation. This approach may be particularly relevant for the diagnosis of coronary microvascular spasm, an endotype historically hampered by the variability of symptom- and ECG-based criteria.

Future studies should explore graded interpretations of ACh testing based on COVADIS criteria components to enhance risk stratification and support more individualized management strategies in patients with ANOCA and MINOCA [23,94,109]. Clinical trials should ideally move towards endotype-specific enrolment, as baseline population heterogeneity of study populations may compromise the evaluation of targeted pharmacological strategies [110].

Furthermore, broader dissemination of expertise, and incorporation of CFT into routine catheterization workflows will be essential to align guideline recommendations and real-world clinical adoption [111,112].

Integrating invasive functional testing with intracoronary imaging may offer a more comprehensive diagnostic approach in the future. In this context, the recent DISCOVER INOCA registry has adopted an invasive physiological assessment together with intravascular imaging to characterize vasomotor phenotypes and morphological features of non-obstructive CAD [113,114]. Of interest, OCT data indicate that plaque erosion and thrombus formation can be observed at spasm sites, and spasm-associated lesions also exhibit a higher prevalence of layered plaques, macrophages, and microchannels, linkable to chronic inflammation and accelerated plaque progression [115,116]. However, whether localized endothelial damage acts as a pre-existing substrate for vasospasm or is a secondary consequence of repeated vasoconstriction remains an open question.

Finally, integrating artificial intelligence (AI) and machine learning into diagnostic workflows holds promise for optimizing CMD assessment using ECG waveforms, multimodal imaging techniques, and automated invasive microvascular assessment [117,118,119]. However, the extension of these models to the predictions and diagnostics of VSA remains largely unexplored and represents an area requiring dedicated future investigation.

## 13. Conclusions

Coronary vasomotor disorders represent a prevalent and prognostically relevant cause of myocardial ischemia in patients with non-obstructive coronary arteries. ACh provocation testing interrogates endothelial integrity and vascular smooth muscle reactivity, enabling the identification of distinct epicardial and microvascular endotypes that cannot be detected by angiography alone. When performed according to contemporary protocols, ACh testing demonstrates a high diagnostic yield and a favourable safety profile, with major complications remaining rare and without evidence of long-term adverse sequelae. Beyond diagnosis, ACh testing carries important prognostic implications and supports mechanism-based therapeutic strategies that improve symptom burden and QoL.

In the era of precision cardiovascular medicine, invasive CFT - including intracoronary ACh provocation testing - should be regarded not as a niche procedure but as a key component of comprehensive evaluation in selected patients with ANOCA and MINOCA, facilitating personalized, pathophysiology-driven care.
